# Physiological Stress Integrates Resistance to Rattlesnake Venom and the Onset of Risky Foraging in California Ground Squirrels

**DOI:** 10.3390/toxins12100617

**Published:** 2020-09-27

**Authors:** Matthew L. Holding, Breanna J. Putman, Lauren M. Kong, Jennifer E. Smith, Rulon W. Clark

**Affiliations:** 1 Department of Natural Resources and Environmental Science, University of Nevada Reno, Reno, NV 89557, USA; 2 Department of Evolution, Ecology, and Organismal Biology, Ohio State University, Columbus, OH 43210, USA; 3 Department of Biology, San Diego State University, 5500 Campanile Drive, San Diego, CA 92182, USA; rclark@sdsu.edu; 4 Biology Department, 5000 MacArthur Blvd., Mills College, Oakland, CA 94613, USA; Laurenkong1@gmail.com (L.M.K.); jesmith@mills.edu (J.E.S.)

**Keywords:** antipredator defense, boldness, integrated phenotype, trade-offs, *Otospermophilis beecheyi*, *Crotalus o. oreganus*

## Abstract

Using venom for predation often leads to the evolution of resistance in prey. Understanding individual variation in venom resistance is key to unlocking basic mechanisms by which antagonistic coevolution can sustain variation in traits under selection. For prey, the opposing challenges of predator avoidance and resource acquisition often lead to correlated levels of risk and reward, which in turn can favor suites of integrated morphological, physiological and behavioral traits. We investigate the relationship between risk-sensitive behaviors, physiological resistance to rattlesnake venom, and stress in a population of California ground squirrels. For the same individuals, we quantified foraging decisions in the presence of snake predators, fecal corticosterone metabolites (a measure of “stress”), and blood serum inhibition of venom enzymatic activity (a measure of venom resistance). Individual responses to snakes were repeatable for three measures of risk-sensitive behavior, indicating that some individuals were consistently risk-averse whereas others were risk tolerant. Venom resistance was lower in squirrels with higher glucocorticoid levels and poorer body condition. Whereas resistance failed to predict proximity to and interactions with snake predators, individuals with higher glucocorticoid levels and in lower body condition waited the longest to feed when near a snake. We compared alternative structural equation models to evaluate alternative hypotheses for the relationships among stress, venom resistance, and behavior. We found support for stress as a shared physiological correlate that independently lowers venom resistance and leads to squirrels that wait longer to feed in the presence of a snake, whereas we did not find evidence that resistance directly facilitates latency to forage. Our findings suggest that stress may help less-resistant squirrels avoid a deadly snakebite, but also reduces feeding opportunities. The combined lethal and non-lethal effects of stressors in predator–prey interactions simultaneously impact multiple key traits in this system, making environmental stress a potential contributor to geographic variation in trait expression of toxic predators and resistant prey.

## 1. Introduction

Predator–prey interactions are often prolonged, dynamic encounters involving an array of traits expressed by both parties. Key traits can include behavioral [1,2,3], morphological [4,5], and physiological features [6,7], creating a multi-dimensional trait space in which natural selection can act [8]. Traits shaped by natural selection in this fashion can be potentiated and/or constrained by phenotypic integration, with sets of traits predisposed to correlations within individuals, populations, or species (for review, see [9]). Integration in this context is related to the concept of behavioral syndromes, which focuses more narrowly on correlated suites of behavior [10]. Phenotypic integration can result from genomic linkage, shared function, shared physiological effectors, and ultimately from correlated selection pressures [11,12]. For example, a behavioral phenotype like boldness while foraging may be favored in resource-limited environments but selected against by predation pressure [13]. In response to predator attacks, other traits such as morphological or physiological defenses are exposed to selection, and their effectiveness may be modulated by behavioral phenotypes [14,15]. Modeling the relationships among distinct traits, as well as their potential physiological underpinnings, can identify both the strength and form of phenotypic integration in antipredator traits [16].

Hormones are common mediators of phenotypic integration. For example, many hormones govern contemporaneous developmental changes during maturation [17,18,19]. Hormones may also induce integration via pleiotropic effects on phenotypically-plastic traits [19,20,21,22]. In vertebrates, glucocorticoids (GCs) are steroid hormones (e.g., cortisol, corticosterone) that mediate a suite of “stress” responses to biotic and abiotic challenges [23,24]. GCs can enact a cascade of changes via the hypothalamic–pituitary–adrenal (HPA) axis over short timescales, ranging from mobilizing energy reserves to depressing immune function [25,26]. GCs are also important in mediating prey responses to predators via two major mechanisms: (1) constraining foraging efficiency [27,28,29] or (2) promoting a surge in GCs which—if chronically imposed—can reduce prey fitness [30,31]. Generally, baseline GC levels are physiological indicators of relative condition or allostatic load, offering a useful tool for monitoring health and reactivity to threats [32,33]. If GCs impact multiple phenotypes expressed during predator–prey interactions, they may constrain the trait combinations exposed to selection and magnify the fitness effects of interactions among predator and prey [34].

Many species of predators use venom to subdue their prey [35]. For prey, avoiding envenomation is a multistep process that can be mediated via behavioral [36,37] and physiological processes [38,39,40]. Behaviors such as awareness, aggression, agility, and deception can help prevent venomous bites or stings, and such behaviors are often accompanied by physiological resistance to the effects of the venom itself [38,39,41,42]. Physiological resistance occurs either by the evolution of reduced susceptibility of venom target tissues [38,40] or via the presence of inhibitor molecules derived from the innate immune system [43,44,45,46]. Phenotypic integration of behavioral and physiological traits in a prey population may emerge from pleiotropic effects of a common underlying cause [47,48]. The GCs, in particular, are a strong theoretical candidate for joint modulation of behavior and venom resistance, given their dual roles in affecting behavior [49] and immune system [25,26]. These effects are expected especially in species for which the baseline GCs of individuals are consistent over time [50]. Such correlations might be adaptive if, for example, those individuals that are more willing to inspect and engage with a venomous predator are also the individuals most resistant to venom, potentially leading to different evolutionarily stable strategies for defense.

Here, we investigated the potential for, and nature of, integration of risk-taking behaviors and physiological resistance to snake venom in a population of California ground squirrels (*Otospermophilus beecheyi*, henceforth “ground squirrels”) hunted by northern Pacific rattlesnakes (*Crotalus oreganus oreganus*, henceforth ”rattlesnakes”). Rattlesnakes are the top ground squirrel predator in many areas [51] and can consume up to 34% of ground squirrel annual reproductive output [52]. Extensive research within and between populations of ground squirrels has revealed diverse anti-snake behaviors that vary ontogenetically within individuals [3,53,54] as well as among populations [53,55]. Similar ontogenetic [56] and population-level [39,57] variation has been observed in venom resistance, measured by either experimental envenomation or assays of serum inhibition of venom enzymatic activity. Most research on ground squirrel venom resistance has involved resistance to the action of snake venom metalloproteinases (SVMP) (e.g., [42,45,57]). The SVMPs generate localized hemorrhage by destroying connective tissue and are thus hypothesized to be “gateway toxins” that facilitate diffusion of venom from the bite site, potentiating downstream lethal effects [42]. They are a major component of *C. o. oreganus* venom by weight and number of isoforms [58,59] and coevolve with geographic variation in ground squirrel resistance [57]. Hence, resistance to SVMP activity in particular is a key trait for surviving interactions with rattlesnake predators.

Exposure to snakes exerts selection to maintain these potentially costly defensive traits: squirrels in areas with high rattlesnake densities express the most effective behavioral and physiological defenses [42,56], and adults express more effective behavioral and physiological defenses than juveniles [3,55,60]. For example, even though tail-flagging is a snake-specific display that deters snakes from striking [1], squirrels adapted to high levels of rattlesnake predation tend to exhibit lower signaling rates, spend less time engaging snakes, and are more hesitant to approach snakes closely than squirrels from areas with low rattlesnake densities (“nonadapted” to snakes) [53,55,61]. Furthermore, boldness and aggression toward snakes has been shown to vary consistently among individual squirrels, indicating that these anti-snake behaviors are associated with individual temperament [62]. The significant body of literature on the causes and correlates of both behavioral responses to snakes and venom resistance in ground squirrels, in combination with repeatable baseline measures of GCs and behavioral traits over time [63,64], raises a clear possibility for long-term correlated selection of these traits. Potential links between behavior and resistance phenotypes within individual ground squirrels, and the mode of such phenotypic integration, have not yet been investigated.

We evaluated two alternative hypotheses for the connection between ground squirrel physiology and multi-trait defenses against snake predation: (1) a “resistance facilitates boldness” hypothesis where venom resistance directly predicts risky behavior during snake encounters or (2) a “shared physiological correlates” hypothesis where venom resistance and risky behavior are integrated via shared physiological mediators but do not directly affect each other. To make these assessments, we jointly quantified three measures of risk-taking in the presence of snake predators (proximity to snake, tail-flagging rate, and latency to feed), physiological resistance to rattlesnake venom, fecal glucocorticoid metabolite (FGM) levels (a validated measure of ground squirrel stress [63]) and body condition (a measure of resource acquisition and energy balance, i.e., fatness) for each individual ground squirrel. Our efforts represent the first joint assessments of physiological venom resistance and behavior within the same individual ground squirrels, elucidating the complex relationship between defensive traits in this model system for studying predator–prey interactions.

## 2. Results

### 2.1. Predictors of Venom Resistance

**Figure 1:**
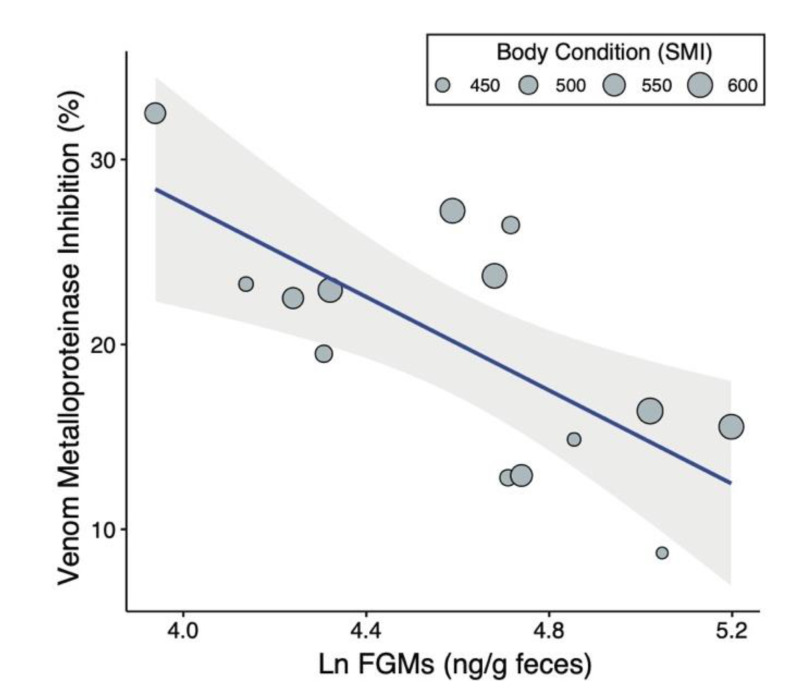
Negative relationship between baseline fecal glucocorticoid metabolites (FGMs) and mean venom metalloproteinase inhibition achieved by squirrel blood serum. Points are sized by squirrel body condition as measured by the scaled mass index (SMI), where squirrels in better body condition tended to show more effective serum venom inhibition.

Overall, ground squirrels with high baseline GCs and in poorer body condition had lower resistance (Figure 1, Model *R*^2^ = 0.57). Higher baseline FGM concentrations predicted lower venom resistance (Table 1). For each 100% increase in FGM concentration, our data reveal a reduction in venom resistance of about 8.2% (back-transforming from the natural log). Assuming this effect size and a causal relationship between corticosterone and venom resistance, and given our sampling of a 51 to 181 ng/g FGM concentration, the potential exists for changes in corticosterone concentrations to reduce venom resistance by 50%. Body condition also predicted venom resistance; ground squirrels in better body condition were significantly more resistant to the effects of rattlesnake venom (Table 1). According to our model, each additional 25 g of relative mass is associated with a 1% increase in SVMP inhibition. On average, squirrels in the best body condition showed 25% more venom inhibition than squirrels in the poorest condition. Sex was not a significant predictor of venom resistance (Table 1), but we had few males in our sample (N = 4 out of 15), so our power to detect sex effects is limited. Taken together, these results suggest a role for an individual’s health and state of stress in levels of physiological venom resistance.

### 2.2. Predictors of Risk-Taking Behaviors

**Figure 2:**
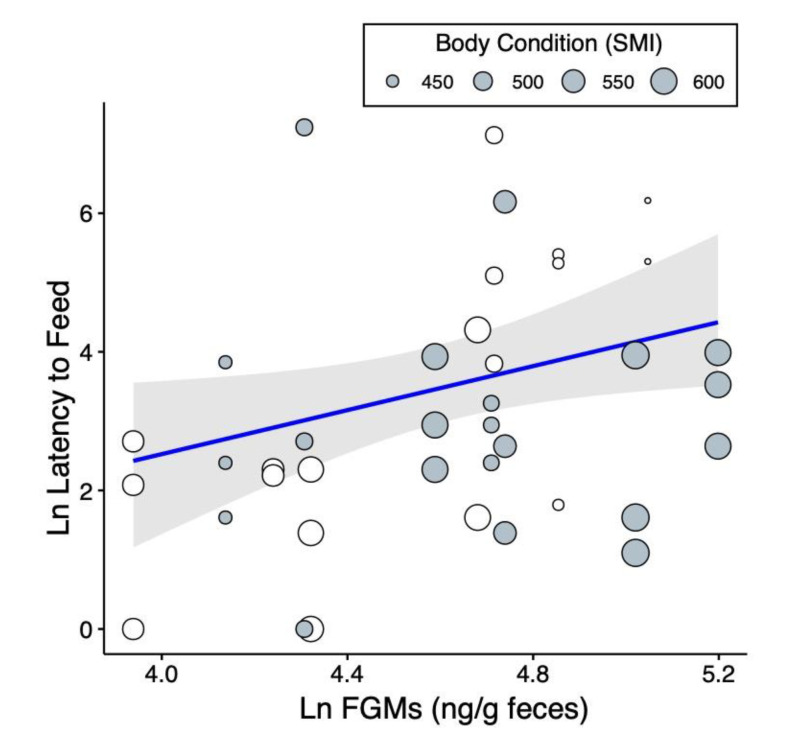
Relationship between ground squirrel baseline fecal glucocorticoid metabolite (FGM) concentration and latency to begin feeding in the presence of a snake. Points are sized by squirrel body condition as measured by the scaled mass index (SMI), where squirrels in better body condition tended to begin feeding sooner. Points stacked vertically indicate repeated measures of latency from the same individual squirrel (vertical stacking is a product of using a single FGM estimate), and points are colored in horizontally alternating white and gray to help identify these repeated measures.

Ground squirrel tail-flagging rates (Table 2) were not significantly related to any of the measured independent variables. The minimum observed proximity to the snake during an encounter was significantly associated only with snake size and snake species (Table 2). Consistent with previous findings [37,65], squirrels showed evidence of altered approach behavior based on the size and species of snake presented. They maintained an average 4% greater distance from the snake for each additional centimeter of snake body size, and squirrels stayed 54.6% further away from rattlesnakes than from gopher snakes. Notably, feeding latency during snake exposure was predicted by the same variables that predicted venom resistance (Table 2, Figure 2). Ground squirrels with higher FGM concentrations waited significantly longer to begin feeding in the presence of a snake. Each 10% increase in FGM concentration corresponded to squirrels waiting 26.4% longer to feed. Body condition also predicted latency to feed, where ground squirrels in better condition began feeding significantly sooner. Each 10-g increase in relative mass predicted a 10% decrease in latency to feed. Repeatability analyses for each behavior revealed significant repeatability of tail-flagging rate (repeatability; *R* = 0.58, *p* < 0.001), proximity to the snake (*R* = 0.32, *p* < 0.001), and for latency to feed (*R* = 0.22, *p* < 0.001).

### 2.3. Integration of Resistance and Behavior

**Figure 3:**
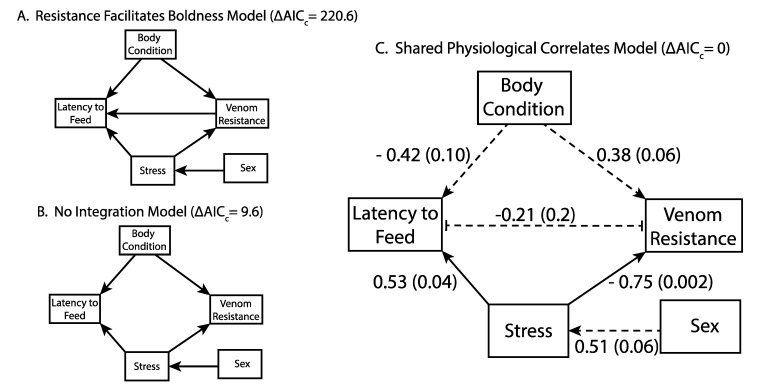
Structural equation models for the interrelationships between California ground squirrel sex, physiological stress and condition, venom resistance, and latency to feed in the presence of a live snake. Models test the hypotheses that resistance facilitates boldness (**A**) directly, that stress does not lead to phenotypic integration of resistance and behavior (**B**), and that resistance and behavior are related via physiological correlates (**C**). In panel (**C**), the solid arrows indicate significant relationships at, α = 0.05 while dashed arrows are non-significant. Numbers next to lines in C are standardized path coefficients with associated *p*-values in parentheses. The standardized path coefficients represent predicted effects in terms of standard deviations of change in each variable, and thus allow comparison of relative variable importance. The line connecting latency to feed and venom resistance is representative of their correlated error structure in this model.

We formulated three separate structural equation models (SEMs) for the relationships among behavior, resistance, and their correlates to model potential causality between FGM concentration, body condition, venom resistance, and latency to feed (Figure 3). The three specified models were similar in structure, except for the relationship between venom resistance and latency to feed, but only one model emerged as the best. Specifically, the shared physiological correlates model (Figure 3C, AIC_c_ = 183.2, ΔAIC_c_ = 0) was significantly better than both the resistance-facilitates-boldness model (Figure 3A, AIC_c_ = 403.8, ΔAIC_c_ = 220.6) and the no-integration model (Figure 3B, AIC_c_ = 192.8, ΔAIC_c_ = 9.6). The improved performance of the shared physiological correlates model supports the hypothesis that stress and body condition independently impact boldness (low latency to feed) and venom resistance, producing an integrated resistance and behavioral phenotype.

The shared physiological correlates model presented in Figure 3 is accompanied by standardized path coefficients, which reflect the relative effects along each path in terms of standard deviations, and thus facilitate comparison of predictor effects among paths (see Figure S1 for raw slope coefficients). The shared physiological correlates model showed non-significant impacts of sex on stress (FGM concentration). However, the *p*-value for this comparison is marginal (*p* = 0.06). Stress remains a significant predictor of resistance (*p* = 0.002), while body condition becomes non-significant at *p* = 0.06. Regardless of their statistical significance, comparing standardized path coefficients indicates that changes of one standard deviation in stress state have larger impacts on resistance than comparable changes in body condition. Stress continued to impact latency to feed (*p* = 0.04) when the dataset consisted of means of behavioral repeated-measures, while the effect of body condition became non-significant (*p* = 0.10). Interestingly, the path coefficient for the relationship between latency to feed and venom resistance was negative in the shared physiological correlates model (−0.21), indicating that more resistant squirrels tend to feed more readily following snake exposure. This suggests that the boldest squirrels should also be more resistant to venom, although there may not be a specific mechanism that links these traits strongly enough to detect a significant correlation in our data.

## 3. Discussion

Our joint assessment of variation in behavioral and physiological adaptations to venomous rattlesnake predators revealed that the same physiological factors predict both a measure of boldness (latency to feed in the presence of a predator) and the ability to inhibit snake venom metalloproteases. Behaviors and resistance have been investigated within and between ground squirrel populations in the past, but our simultaneous assessment within the same individual squirrels allowed us to test for potential integration of these traits via shared effectors. The difficulty of collecting these data resulted in a limited sample size of individual squirrels. Nevertheless, different analytical approaches indicated that stressed ground squirrels are both less bold and less resistant to venom, findings consistent with our understanding of the molecular basis of venom resistance [43,45] and the impacts of glucocorticoids on immune function and feeding behavior in other systems [25,26]. The correlations between FGM concentrations and behavior and venom resistance raised the possibility that ground squirrel boldness and resistance are integrated phenotypes.

Structural equation modeling of relationships among stress, venom resistance, and behavior supported the shared physiological correlates hypothesis for integration of behavior and venom resistance. Our findings suggest that corticosterone is a likely candidate for hormonal integration of multiple aspects of predator avoidance in these squirrels. We do caution that the power of SEMs to appropriately discriminate among models is limited with small sample sizes like ours [66], and therefore the SEM results are the most tenuous of our findings. Although our SEM-based conclusions are bolstered by the shared impact of stress shown in the individual linear models (e.g., Table 1 and Table 2), issues with power are likely reflected by the lost statistical significance of the relationship between sex and stress, and between body condition and venom resistance, in the top SEM model. Confirmation of predictions of the shared physiological correlates hypothesis will require future work in this system with larger sample sizes, experimental manipulations, and repeated measures of both FGMs and venom resistance within individuals. Such studies may reveal an additional key context for hormonal integration of anti-snake defenses, such as distance from refuges (e.g., burrows, [67]), availability of promontories (e.g., elevated rocks or logs, [68]), foraging group size [60], and the presence of genetic relatives [69] or other safety cues [70].

Integration of behavior and venom resistance has important implications for predator–prey coevolution. Hormonal control of both phenotypes implies that the multivariate anti-snake trait space of ground squirrels is constrained. Thus, ground squirrel phenotypes are expected to range between individuals with correlated traits (syndromes) that vary along a continuum of “low risk tolerance and high susceptibility to venom” versus “high risk tolerance and low susceptibility to venom” phenotypes. When only certain phenotypic combinations are exposed to natural selection, canalization of phenotype space can be reinforced by the evolution of strengthened integration [71]. Over the lifespan of an individual ground squirrel, the presence and direction of boldness-resistance integration have intuitive benefits. Namely, a healthy squirrel is more physiologically resistant to snakebite, and may take more risks to acquire resources. On the other hand, a squirrel that is fighting infection, healing a wound, or investing heavily in growth or reproduction will be less resistant to venom, and more risk-averse.

Over evolutionary timescales, selection from snake predators can reinforce this integration mechanistically. Evolutionarily stable strategies have emerged as a product of this type of integration, with prominent examples being metabolism–growth relationships in *Uta stansburiana* lizard morphs [72], carnivorous and omnivorous morphs in spadefoot toads (*Spea*) [73], and morphology–growth rate integration in *Pinus* trees [74]. In ground squirrels, assuming geographic variation exists in average stressfulness of different environments, it is easy to imagine how geographic mosaics of trait expression [75] could occur due to stress-induced phenotypic plasticity alone. Alternatively, patterns of integration may change over the course of an individual squirrel’s lifetime as body condition and stress levels change. For instance, the associations we measured could be the result of a reproductive trade-off such that adult female squirrels that invested in reproduction experienced nutritional stress and decreased body conditions, leading to changes in venom resistance and foraging behaviors. We did not record the reproductive status of the squirrels, which could have further explained variation in our models as previous studies have shown that maternal squirrels with pups are more aggressive toward snakes and are more willing to approach them closely compared to males or non-maternal females [76,77]. More work on how these phenotypic relationships vary among individuals and change over their lifetime is clearly warranted.

The serum-based resistance of ground squirrels appears to be derived from the innate immune system, as it is driven by various members of the immunoglobulin-superfamily (IgSF) [43,45], and innate immunity can be depressed by stress [78,79]. The link between ground squirrel venom resistance and stress has important implications for previous and future studies of population-level variation in both predator venom and prey venom resistance. A positive relationship between serum-based venom resistance and estimates of local snake density has been previously documented and interpreted as a response to selection from snake predators [39,42]. If localities with high snake densities are less stressful to squirrels (perhaps because they are also habitats less impacted by human development) [63], phenotypic plasticity could offer a second potential explanation for patterns of observed variation. More recently, ground squirrels from several sites of high snake density were shown to vary in venom resistance, but higher site elevation was associated with lower venom resistance [57]. There are numerous examples of elevation or latitude impacting baseline corticosterone levels in vertebrates [80,81]. Anthropogenic effects, growing season duration, and average temperature might be expected to vary with elevation in a manner that drives a positive relationship between corticosterone levels and elevation. As such, it is possible that some degree of population-level variation in resistance observed by Holding et al. [57] was phenotypic plasticity associated with stress. Future work on rattlesnake–squirrel coevolution should include plasma or fecal sampling so that corticosterone concentrations can be considered as a covariate in evolutionary analyses.

We used close proximity to snakes, tail-flagging rate, and latency to feed as three separate measures of boldness, which we defined as willingness to take risks during a predator presentation test [82]. Yet, we did not find that these three behaviors were similarly impacted by the same effectors, implying that they represent functionally different traits for squirrels [83]. For instance, approaching snakes closely may allow squirrels to more accurately assess the threat posed by the snake, tail-flagging modifies snake hunting behavior [1], and feeding latency associates with resource acquisition. In all, we found that only individual variation in resource acquisition was affected by baseline corticosterone levels and, to a lesser degree, body condition. Previous studies have shown that squirrels modify tail-flagging and aggression towards snakes (including proximity) based on various intrinsic and extrinsic factors including snake size, snake species, squirrel sex, squirrel reproductive status, and distance from home burrow [37,76,77]. We were able to account for some of these factors in our analyses and found that snake size and species influenced the willingness of squirrels to approach snakes closely (they were more bold in the face of small snakes and non-venomous snakes). We did not document squirrel reproductive status and precise distance from the home burrow system, but previous work has shown that maternal females are more willing to engage snakes and that squirrels are more defensive near their home burrows [76,77]. We tested squirrels in the general vicinity of their home burrow system, based on our observations of their burrow use, but including the above factors as co-variates could have further improved our models. Nonetheless, our study provides evidence that aspects of squirrel physiology, including resistance to rattlesnake venom, are intrinsically linked with at least some risk-taking behavior.

Associations between physiological health and behavior are indicative of the presence of different coping styles (a type of phenotypic integration), in which neuroendocrine characteristics underlie behavioral responses to environmental stressors [84]. The “risky foraging” we measured could be modulated by individual state—an intrinsic characteristic of the individual expressing the behavior—through various mechanisms [85]. The asset-protection hypothesis states that risk-taking should decrease with increasing reproductive value (e.g., better body condition) because of the increased importance of protecting that asset [86]. Our results do not support this hypothesis, as squirrels in better condition had lower latencies to feed under the risk of predation. Instead, our results support the state-dependent safety hypothesis, which assumes a positive-feedback mechanism whereby individuals with higher energy reserves are better at coping with predators, and subsequently maintain high levels of boldness [87]. Several studies show that individuals in good body condition are willing to take greater risks because they are better at escaping from predators than individuals in a poor condition [88,89]. Although we do not have data on whether well-fed squirrels are better at escaping snake strikes, future work could assess this along with whether differential access to resources maintains differences in behavioral responses in this species [27,28,29].

## 4. Conclusions

Stressed squirrels are worse at inhibiting rattlesnake venom and could have lower innate immunity [90]; therefore, they are more risk-averse compared to squirrels with lower corticosterone levels. We specifically found that fecal corticosterone concentrations negatively correlate with latency to feed: stressed squirrels were hesitant to approach a food source when a predator was present. Thus, stress appears to reduce opportunities for resource acquisition, which could subsequently lower body condition, contributing to the above-mentioned feedback loop. Overall, it seems that while glucocorticoids help modulate predator avoidance behavior in ground squirrels, they also incur costs that are likely to have other negative fitness effects. Our current study extends previous knowledge of the links between ground squirrel physiological health and body condition [63]. Although physiological and fitness correlates exist for related mammalian species [91], future work will be required to explicitly assess the direct effects of the integrated response documented in our study on survival and reproduction in ground squirrels. Hormonal organization of integrated phenotypes in venom-resistant species may provide the flexibility to express alternative, costly strategies for resource acquisition dependent on the present conditions impacting an individual.

## 5. Materials and Methods

### 5.1. Study Site and Squirrel Trapping

California ground squirrels were studied from May to July 2013 at the Blue Oak Ranch Reserve (BORR), Santa Clara County, California. Here, ground squirrel pups typically emerge from natal burrows in April/May, after which they are no longer dependent on mothers for nutritional support [92]. Rattlesnakes also begin hunting in squirrel colonies around this time. At the beginning of the field season, we trapped squirrels to dye-mark their fur for individual identification. Following the methods of Owings et al. [61] we captured squirrels using Tomahawk traps baited with black oil sunflower seed, and sedated them with 40 mg/kg of Ketamine through injection into the hind leg muscle. We marked anesthetized squirrels with Nyanzol pelage dye for short-term identification and metal ear tags for long-term identification, weighed and sexed them, and measured their body length, tail length, and hindfoot length. Mass and body length measurements were used to calculate body condition using the scaled mass index (SMI) [93]. Last, we collected a blood sample via cardiac puncture and allowed the blood sample to clot on ice overnight, followed by clot removal and the collection of remnant serum. After processing, we kept squirrels captive until they regained normal movement, and then released them back at the point of capture within the same day. Experimental trials and collection of fecal samples and blood samples (see below) did not occur until at least a week after this initial processing. We did not record reproductive status of squirrels, but all trapping occurred after pups had emerged from the natal burrows and were feeding on vegetation (i.e., females were no longer lactating and pups were no longer dependent on mothers for milk [64]). All protocols complied with the policies of San Diego State University Institutional Animal Care and Use Committee (APF 10-09-025C, Approval Date: 9 March 2013).

### 5.2. Squirrel Risk-Taking Behaviors

We scored ground squirrels for three measures of boldness in the presence of snake predators, either venomous rattlesnakes or non-venomous gopher snakes (*Pituophis catenifer*). In this context, we follow the definition of boldness proposed by Réale et al. [82], which broadly refers to risk-taking, and we specifically measured risk-taking behaviors during a predator presentation test (a boldness assay proposed by [82]). We habituated marked squirrels to return to a specific location near their home burrows by feeding them with sunflower seed at that location (henceforth called the bait station). We placed a small handful of seed within a metal ring near their burrow at least once daily. Because previous research has shown that distance from home burrow (either within or away from home burrow system) affects how squirrels behaviorally respond to snakes [65,76,77], we attempted to test focal squirrels within 20 m of their home burrow system, but never closer than 1 m to a burrow entrance. However, we did have three squirrels in our dataset for which we were unable to determine home burrow location. At each bait station, squirrels were presented with either a live tethered rattlesnake or gopher snake, and their behavioral responses were recorded using handheld video cameras (Model DCR-SR 85, Sony Corporation of America, New York, NY USA). Sunflower seeds were present as a food resource within the bait station during these trials. Trials ended once a squirrel walked away from the bait station.

We began observations of squirrels at approximately 07:00 a.m., before snakes emerge from their nighttime refuges [94], so we are confident that we were able to determine whether squirrels had encountered a snake during their day of testing and excluded these individuals from this study. Field observations were conducted from hunting blinds approximately 15 m away from the bait station. We tethered rattlesnakes using a modified version used previously [3]. We used rubberized twist ties to secure gopher snakes to a 5 × 60 cm wooden plank that was staked into the ground. We presented each squirrel with up to one rattlesnake and one gopher snake in a random order, separated by at least 24 h between tests. Since ground squirrels typically encounter snakes daily [94], we are confident in the realism of the time frame used for snake presentations. We used a total of 12 snakes for these presentations: eight adult northern Pacific rattlesnakes (*C. C. o. oreganus*; snout-vent length: 55.0–96.0 cm, mean ± SE = 81.5 ± 5.2 cm) and four adult Pacific gopher snakes (*P. catenifer*; snout-vent-length: 88.0–103.0 cm, mean ± SE = 96.3 ± 3.2 cm). All snakes were short-term captive adult males large enough to prey on squirrel pups and were initially captured at our field site.

From video recordings, we identified all marked squirrels that approached and interacted with the snake. We quantified two salient behaviors that have been previously documented to vary individually and by population in ground squirrels: the closest approach to the snake and tail-flagging [53,61,62]. Based on this previous literature, we assumed that approaching snakes closely and tail-flagging at lower rates represent more risky or bold behaviors than the opposite, because snakes are more likely to strike squirrels that come in close contact and tail-flagging generally deters snakes from striking [1]. We also quantified latency to feed at the bait station after sighting the snake because past research in other systems have used feeding latency as a robust measure of boldness [95,96].

Upon seeing a snake, squirrels exhibit stereotypical behavior by assuming a fixed orientation posture, termed elongate investigative posture (EIP) [53]. We assumed that squirrels were unaware of the snake until they exhibited this behavior. We defined latency to feed as the time between exhibiting EIP and feeding at the bait station. We measured the closest proximity to the snake by taking a screen shot and using ImageJ to estimate distance within video stills. We used our knowledge of squirrel body lengths, tail lengths, or the width of the bait station as references of known distance for these analyses. We only measured distance if we had a full lateral view of the squirrel. Tail-flagging, waving of the tail from side-to-side, is a signal of snake detection and of squirrel vigilance [1,37]. We divided the number of tail-flagging bouts by the encounter duration (the length of time of the squirrel-snake encounter) to determine the tail-flagging rate. Higher tail-flagging rates associate with squirrels’ ability to initiate an evasive leap away from snakes, indicating a positive relationship with vigilance and arousal [42]. Overall, bolder (less risk-averse) squirrels should exhibit low latencies to feed, close proximities to snakes, and/or low tail-flagging rates.

### 5.3. Fecal Glucocorticoid Metabolites

To assess the adrenocortical activity of squirrels, we collected feces from trapped squirrels and quantified fecal glucocorticoid metabolites (FGMs). Squirrels were trapped repeatedly throughout the field season and fecal samples were collected from 0 to 5 h after squirrels had been trapped. This length of time is not long enough for glucocorticoids associated with trap stress to manifest in feces in this species. A recent validation of the methods used in this paper showed that FGM levels peaked approximately 51.6 ± 9.7 SE h after injection with ACTH [63]. Repeated samples of squirrel feces were taken more than 48 h apart. For samples taken two days apart (N = 10), we have no evidence for later samples having higher FGMs than earlier samples: the average difference in FGMs between samples taken two days apart is −5.81 ± 13 SE ng/g. Therefore, we consider all fecal samples collected to represent baseline FGM values. Although it only occurred rarely, any feces that were mixed with urine were discarded. Fecal samples were stored in liquid nitrogen in the field and later transferred on dry ice to Mills College where they were frozen at −80 °C prior to the extraction of FGMs. In all, we collected 2–11 samples per individual squirrels over the 2013 field season.

In the laboratory, fecal samples were freeze-dried using a lyophilizer (LabConco, Kansas City, MO, USA) for 18–20 h to control for water content. Freeze-dried samples were homogenized using a mortar and pestle. In duplicate, 0.050 g (± 0.001 g) of sample was suspended in 1 mL of 90% aqueous methanol (*v*/*v*) in sterile cryotubes and placed on a multivortexer (VXR basic Vibrax^®^, IKA^®^ -Werke GmbH & Co., Staufen, Germany) orbital shaker at 1450 rpm for 30 min (Sheriff et al., 2012). Well-mixed samples were placed in a cooled centrifuge at 1500 rpm for 30 min. Collected supernatant was frozen at −80 °C until analysis.

We used an enzyme-linked immunosorbent assay after diluting samples with buffer per instructions of the kit (ELISA Kit #500655, Cayman Chemical, Ann Arbor, MI, USA). The primary antibody of this assay is raised against corticosterone, and has a cross-reactivity of 100% with this steroid and of 7–11% with 11-deoxycorticosterone and 11-dehydrocorticosterone, respectively. It has a cross-reactivity of less than 1% with other steroids (e.g., progesterone, cortisol, aldosterone, testosterone). The intra-assay coefficient of variation (CV) was 7.0 ± 0.4% (mean ± standard error) and the inter-assay CV was 17.7 ± 7.0% (N = 6 plates).

### 5.4. Serum Inhibition of Venom Acitivty

We used serum inhibition assays, as described elsewhere [57,97], to quantify serum-based resistance to SVMP activity in 18 different ground squirrel individuals. Briefly, we measured the gelatinase enzymatic activity of venom collected from sympatric Blue Oaks Ranch Reserve rattlesnakes (*C. o. oreganus;* 5 individuals) using the EnzChek Gelatinase/Collagenase Assay Kit (Life Technologies, Carlsbad, CA, USA). We followed product protocols, except that the gelatin substrate was diluted to a concentration of 1: 100 and 0.3125 ng of venom was used in each test well of the 96-well, flat, white microplate (Corning, Inc., Corning, NY, USA). We measured gelatinase activity as fluorescence intensity in relative fluorescence units (RFU) every 1.5 min using a FLUOstar Omega microplate reader (BMG Labtech, Ortenberg, Germany). Venom activity was measured as the slope (RFU/min) from the linear part of the reaction (1.5–18 min). Larger slopes indicate that the venom was degrading the substrate more quickly, and hence that it has higher SVMP activity and, if serum was present, avoided serum SVMP inhibitors. Previous work in *C. o. oreganus* has directly linked gelatinase activity as measured by this reagent kit to SVMPs in the venom. We measured baseline SVMP activity from the venom of five individual snakes in this manner, followed by remeasurement of SVMP activity of the same venom following 30 min of incubation with 18 μL of serum (5 mg/mL) collected from each sampled ground squirrel. All baseline and serum inhibition trials were run in triplicate and the average slope was taken for analysis. Squirrel serum contains inhibitor proteins that confer venom resistance [43,45]. Therefore, the percent reduction in venom activity from its baseline activity following incubation with squirrel serum provides a functional measure of serum-based venom resistance. Each ground squirrel’s serum was tested separately with all five individual snake venoms to provide separate assays of inhibition. Therefore, the average percent inhibition of these five venoms by each squirrel provides a resistance measure that incorporates variation in venom composition of local rattlesnakes.

### 5.5. Statistical Analyses

All raw data used herein are provided as supplementary information. To justify taking an average of repeated samples for our baseline FGM estimate for downstream analyses, we estimated the repeatability of FGMs within individual squirrels using the rptR package [98] in R. We used the LMM method and built a model of baseline FGM levels that accounted for ground squirrel mass, sex, Julian day, hour of the day, and time since snake exposure, and found ground squirrel FGM levels to be significantly repeatable within individuals (N samples per individual = 2–11, *R* = 0.401, *p* < 0.0001). High intra-individual repeatability of FGMs in California ground squirrels has also been confirmed by Hammond et al. [63].

We modeled venom resistance using a general linear model (GLM using the lm function in base R) in N = 15 ground squirrels for which we quantified baseline FGM levels, body condition [93], and sex, and provided partial *R^2^* values for each model term using the rsq.partial function from the rsq package [99]. We used the average percent inhibition for each ground squirrel as the dependent variable, with baseline FGM concentrations, body condition, and sex as fixed factors. FGM concentrations were natural-log transformed to meet the assumption of normality. Resistance values for one ground squirrel were consistently negative in the five replicates, indicating its serum increased venom activity rather than inhibited it, and placing mean venom resistance for this animal more than three standard deviations below the overall average (Figure S2). Because no other squirrel’s serum in our sample potentiated venom activity, we suspected potential sample contamination. We accordingly removed this animal from all presented analyses, resulting in a sample size of 14 squirrels.

We used linear mixed-effects models (LMM) to predict behaviors in 39 trials involving N = 14 individual squirrels for which we also measured venom resistance, body condition, and FGMs. We used LMM, as implemented in the lme function of the nlme R package [100] to model each of the three behavioral responses, and provide full model and partial *R^2^* values using the r2beta function in the r2glmm package [101] in R. Tail-flagging rate was square-root transformed, while proximity to the snake and latency to feed were natural-log transformed. Each of these transformed variables were modeled in separate LMMs as the dependent variable, with snake species, snake body size (SVL), squirrel sex, squirrel body condition, and mean FGM concentration as fixed factors. Squirrel ID was included as a random effect to account for repeated behavioral observations on the same individual. Shapiro–Wilks tests were used to assess normality of the residuals in both models, and linear relationships among variables were assessed by visualizing the residual vs. fitted values for each model. We also used the rptR package to assess individual repeatability for each of these behaviors, using a larger sample of 51 total behavioral observations obtained in this population.

We modeled the among-variable relationships and potential for phenotypic integration in latency to feed and venom resistance using structural equation models as implemented in the piecewiseSEM R package [102,103]. In order to specify models where not all responses were repeated measures (e.g., venom resistance and covariates of FGM concentrations), we took the mean of latency to feed for each ground squirrel individual so that the models could be set up as multivariate general linear models. Previous work showed that sex is predictive of FGM concentrations in ground squirrels [63], so we also included it in formulated models as a conditioning variable. Each SEM was composed of three linear models specified by the lm() function in R, where natural-log transformed latency to feed, venom resistance, and FGM concentrations (stress) are dependent variables in the three respective models. In the “resistance facilitates boldness” model, a causal relationship is implied where ground squirrel venom resistance impacts latency to feed directly. In the ”shared physiological correlates” model, venom resistance and latency to feed are specified to have correlated errors, but no causal relationship between one another (i.e., both are correlated due a shared effector, such as stress or body condition). In a third model, all relationships remain the same as for the shared physiological correlates model, except that latency to feed and venom resistance are directly separated, and therefore specified as not impacting one another or having correlated errors (i.e., there is not phenotypic integration; henceforth ”no integration model”). We compared the three models using sampled-size corrected Akaike Information Criterion (AIC_c_), where we considered ΔAIC_c_ > 2 to indicate significantly poorer model performance compared to the model with the lowest AIC_c_ [104].

## Figures and Tables

**Table 1:** Linear model explaining ground squirrel venom resistance measured by serum inhibition of snake venom metalloproteinase activity. This model explained *R^2^* = 57% of the variation in resistance. Bold font indicates significant effects.

Model Terms	Coefficient	Std. Error	*t*-Value	*p*-Value	Partial *R*^2^
Intercept	51.41	20.46	2.51	0.031	N/A
Ln(Corticosterone)	−11.76	3.82	−3.08	0.012	0.43
Body Condition	0.04	0.02	2.24	0.049	0.17
Sex (Ref. = Male)	−2.90	3.18	−0.91	0.382	0.03

**Table 2:** Linear mixed-effects model results explaining ground squirrel risk-taking behaviors of (i) tail-flagging rate, (ii) proximity to the snake’s position, and (iii) latency to feed following the presence of a snake. Squirrel ID is included as a random effect in all models to control for repeated measurements from the same individual, and significance of the random effect was tested using likelihood ratio tests. Significant fixed factors are in bold.

Behavior	Model Terms	Coefficient	Std. Error	*t*-Value	*p*-Value	Partial *R*^2^
i. Tail-flagging Rate	Intercept	0.73	5.20	0.14	0.890	N/A
*R*^2^ = 16%	Snake Species ^a^	0.12	0.31	0.39	0.703	0.003
	Snake Size	0.00	0.01	0.14	0.887	0
	Squirrel Sex ^b^	−0.07	0.78	−0.10	0.926	0.001
	Body Condition	−0.01	0.00	−1.19	0.262	0.105
	Ln(Corticosterone)	0.76	0.93	0.82	0.430	0.059
	Squirrel ID	Variance = 0.60	SD = 0.77	10.1	0.002	N/A
ii. Proximity to Snake	Intercept	−0.96	3.47	−0.28	0.784	N/A
*R*^2^ = 28%	Snake Species ^a^	−0.79	0.25	−3.13	0.005	0.173
	Snake Size	0.04	0.01	3.08	0.005	0.176
	Squirrel Sex ^b^	−0.43	0.51	−0.85	0.415	0.044
	Body Condition	0.00	0.00	−0.21	0.838	0.003
	Ln(Corticosterone)	0.21	0.60	0.34	0.738	0.008
	Squirrel ID	Variance = 0.38	SD = 0.62	4.2	0.040	N/A
iii. Latency to Feed	Intercept	−3.89	5.39	−0.72	0.477	N/A
*R*^2^ = 33%	Snake Species ^a^	−0.48	0.63	−0.76	0.453	0.018
	Snake Size	0.02	0.03	0.53	0.601	0.009
	Squirrel Sex ^b^	−0.60	0.73	−0.83	0.428	0.021
	Body Condition	−0.01	0.00	−2.24	0.049	0.136
	Ln(Corticosterone)	2.50	0.84	2.98	0.014	0.218
	Squirrel ID	Variance = 2.7	SD = 1.6	−12.1	1	N/A

^a^ Rattlesnake is the reference level for snake species, ^b^ female is the reference level for sex. SD = Standard deviation.

## Supplementary Materials

The following are available online at https://www.mdpi.com/2072-6651/12/10/617/s1, Figure S1: Duplicate representation of the shared physiological correlates model where path coefficients are shown as raw slope coefficients (unstandardized) for each path, Figure S2: Distribution of serum-based venom metalloproteinase inhibition values (% inhibition) for 17 California ground squirrel individuals. The point at −11.5% represents a high leverage outlier, and was removed from analyses presented in the manuscript title., RawData.xlsx: Raw data from the study.
