# Supplementary material for: Physiological Stress Integrates Resistance to Rattlesnake Venom and the Onset of Risky Foraging in California Ground Squirrels
Source: Toxins (Basel). 2020 Sep 27;12(10):617. doi: 10.3390/toxins12100617 (PMC7601495; doi:10.3390/toxins12100617)
Supplement: Supplementary file 1 [file toxins-12-00617-s001.pdf]

Supplementary Materials: Physiological Stress Integrates Resistance to Rattlesnake Venom and the Onset of Risky Foraging in California Ground Squirrels

Matthew L. Holding, Breanna J. Putman, Lauren M. Kong, Jennifer E. Smith and Rulon W. Clark

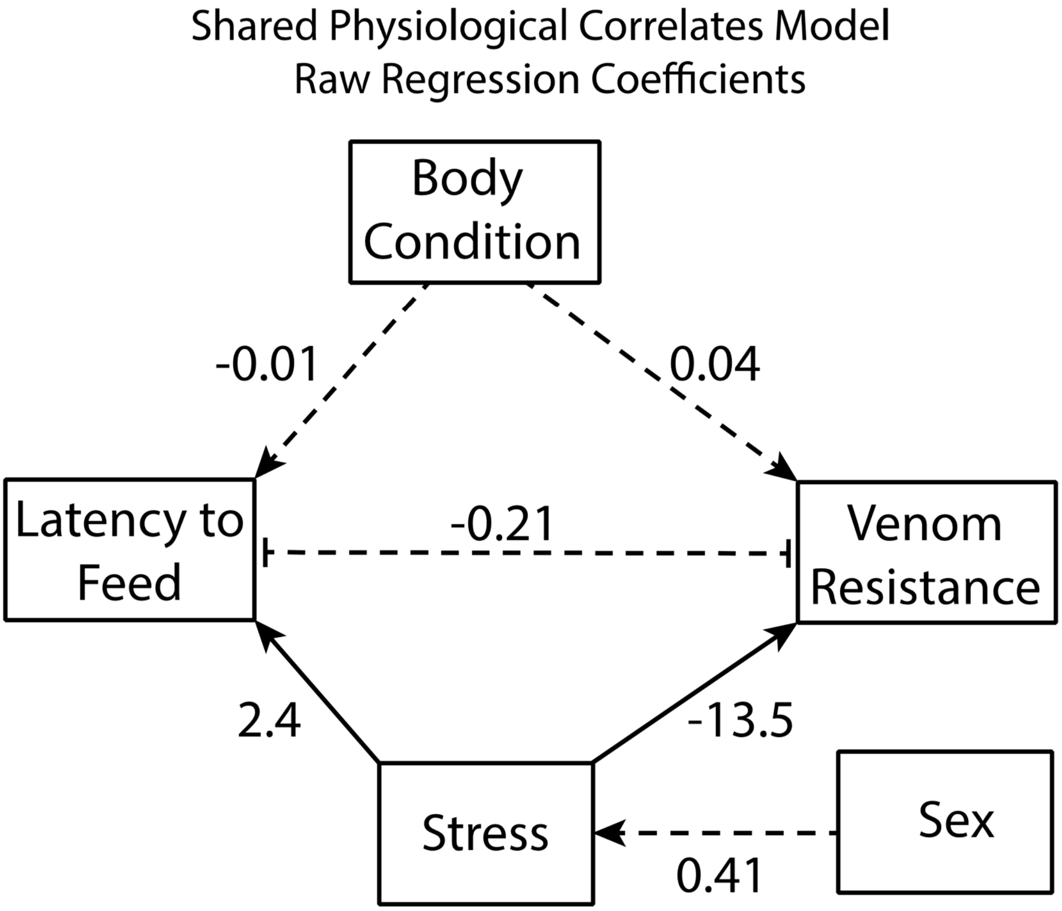

Figure S1. Duplicate representation of the shared physiological correlates model where path coefficients are shown as raw slope coefficients (unstandardized) for each path.

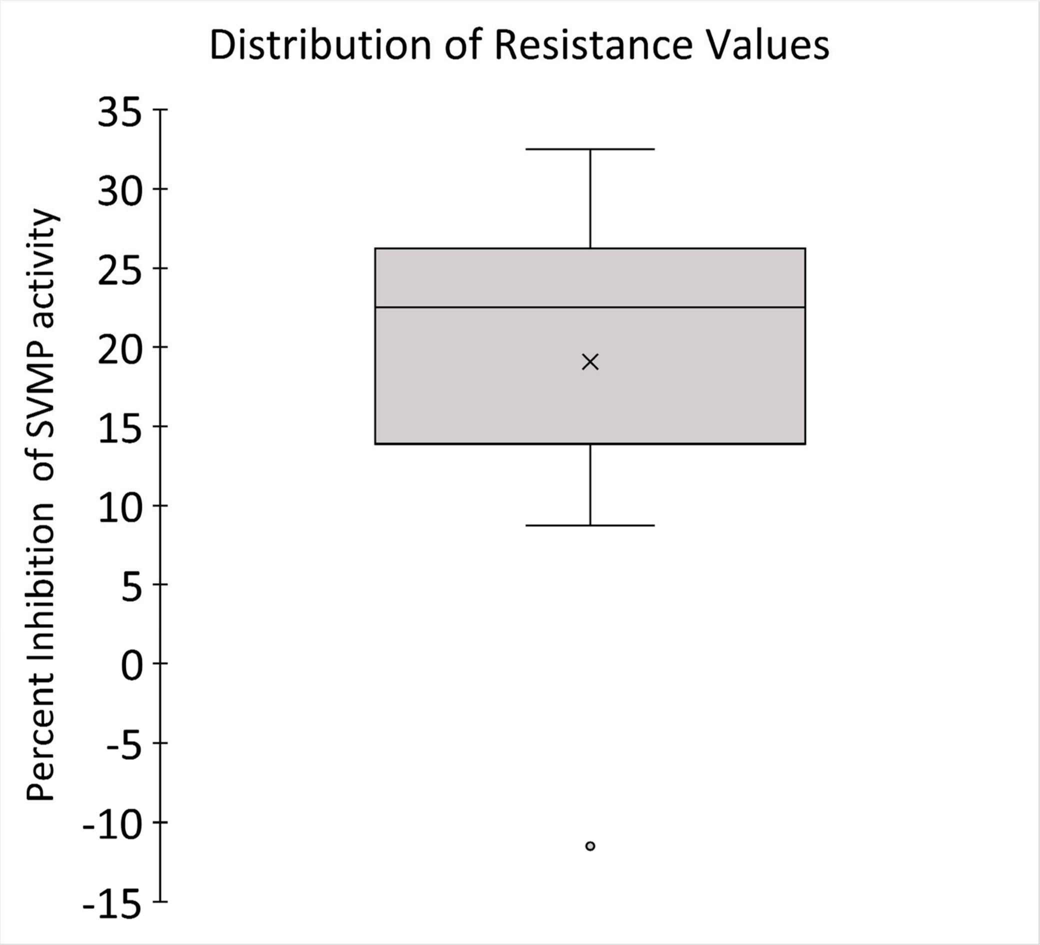

Figure S2. Distribution of serum-based venom metalloproteinase inhibition values (% inhibition) for 17 California ground squirrel individuals. The point at −11.5% represents a high leverage outlier, and was removed from analyses presented in the manuscript.
